# Prescription Ingredients in Skin‐Lightening Products Found Over‐the‐Counter Post–Hydroquinone Ban: Cross‐Sectional Study of 70 Stores in the Twin Cities

**DOI:** 10.1111/jocd.70005

**Published:** 2025-03-03

**Authors:** Puneet Arora, Caroline Brumley, Sara Hylwa

**Affiliations:** ^1^ Department of Dermatology Park Nicollet Health Services Minneapolis Minnesota USA; ^2^ University of Minnesota Medical School Minneapolis Minnesota USA

To the Editor,

Skin‐lightening or “bleaching” is a common cosmetic practice worldwide and reflects ongoing effects of colorism, which refers to the discrimination against and parity of opportunities available to darker‐skinned individuals compared to those with lighter skin across different ethnic and racial groups [1]. Long‐term exposure to ingredients in skin‐lightening products (SLPs) like hydroquinone and prescription‐strength topical corticosteroids (PSTCs) can lead to irreversible skin damage. Hydroquinone use has been associated with exogenous ochronosis, periorbital hyperpigmentation, vitiligo‐like lesions, and contact dermatitis [2]. Side effects of PSTCs include skin atrophy, striae, steroid‐induced acne, and possible systemic effects with long‐term use of high‐dose topical steroids [2]. Unfortunately, most consumers are unaware of these risks. This matter disproportionally affects patients with skin of color and has been labeled as a public health issue [2]. As part of the CARES Act enacted in 2020, the sale of hydroquinone in over‐the‐counter (OTC) products was effectively banned as SLPs containing it now require approval from the US Food and Drug Administration (FDA). However, patients continue to present to clinics with regular use of these products [3].

Prior publications have found widespread availability of OTC PSTCs across multiple US cities. There has been a call for further research assessing the prevalence of other potentially harmful prescription‐only medications sold OTC, including SLPs [4, 5]. The goal of this study was to ascertain the availability of SLPs containing prescription‐level ingredients in the Twin Cities 3 years post banning of OTC hydroquinone.

In this cross‐sectional study, we investigated 70 stores across Minneapolis and St. Paul, MN, and the surrounding areas. The list of stores was generated by internet searches for “ethnic stores” and “imported skincare.” All stores advertising a specialty in the sale of products originating from countries outside of the United States were included. Investigators visited each store and quantified the total number of SLPs, defined as any product claiming to have skin lightening properties, along with the number containing prescription‐level ingredients, notably hydroquinone and/or PSTCs. Store country/region of origin was also documented as self‐advertised by the business owners.

Of the 70 stores investigated, 32 (45.7%) sold SLPs, 7 (21.9%) of which sold hydroquinone‐containing products and 11 (34.4%) with PSTCs (Table 1). 57.1% (4/7) of hydroquinone products and 63.6% (7/11) of PSTCs were found at Hmong or Somali stores, two communities of local significance, which made up only 28.6% of total stores visited. While 88.9% (8/9) of South Asian stores sold SLPs, none contained hydroquinone or PSTCs. However, two of these stores did sell hydrogen peroxide‐containing SLPs. Notably, only 2/32 (6.25%) stores carrying SLPs were included in a recent report by the Minnesota Department of Health, suggesting that many of the SLPs identified here have not been previously evaluated [6].

**TABLE 1:** Skin‐lightening products in the Twin Cities.

Ethnicity	SLPs sold/stores visited, *n*/*N* (%)	Hydroquinone prevalence, *x*/*n* (%)^a^	PSTC prevalence, *x*/*n* (%)^a^
Total	32/70 (45.7%)	7/32 (21.9%)	11/32 (34.4%)
African	12/20 (60.0%)	4/12 (33.3%)	7/12 (58.3%)
Somali^b^	8/14 (57.1%)	3/8 (37.5%)	5/8 (62.5%)
East Asian	10/26 (38.5%)	3/10 (10.0%)	4/10 (40%)
Hmong^b^	2/4 (50%)	1/4 (25%)	2/4 (50%)
South Asian^c^	8/9 (88.9%)	0/8 (0%)	0/8 (0%)
Hispanic	2/14 (14.3%)	0/2 (0%)	0/2 (0%)
Various	0/1 (0%)	—	—

Abbreviations: PSTC = prescription‐strength topical corticosteroids, SLPs = skin‐lightening products.

^a^ Prevalence among stores selling SLPs.

^b^ Ethnic communities of local significance.

^c^ Two stores additionally sold hydrogen peroxide‐containing skin‐lightening creams.

Despite recent FDA rulings, imported SLPs containing hydroquinone are highly available in the Twin Cities, illustrating a continued demand for such products. These findings call into question the possibility of similar availability nationwide. SLPs containing PSTCs are also widely available, consistent with previous findings [4]. Dermatologists have a responsibility to understand the history of skin bleaching, current practices in one's community, and the mental and physical impacts of such practices [1]. Patients purchasing products from these stores are likely unaware of the harms associated with long‐term hydroquinone and PSTC use, as these products are available legally OTC in many countries. Awareness that these ingredients remain accessible OTC despite policy changes may lead to earlier detection or prevention of long‐term side effects in affected patients.

There are limitations to this study. The prevalence of these products may be under‐reported as SLPs were included only if they were directly accessible by the investigator or upon request to store staff. Additionally, the prevalence of such products reported here is limited to the Twin Cities and may not be representative of other populations. Further research is needed to assess the availability of skin lightening products in other geographic areas.

## Ethics Statement

The authors have nothing to report.

## Conflicts of Interest

The authors declare no conflicts of interest.

## Data Availability

The data that support the findings of this study are available on request from the corresponding author. The data are not publicly available due to privacy or ethical restrictions.

## References

[1] K. Daftary , N. S. Krishnam , and R. V. Kundu , “Uncovering the Roots of Skin Bleaching: Colorism and Its Detrimental Effects,” Journal of Cosmetic Dermatology 22, no. 1 (2023): 337–338, 10.1111/JOCD.15049. 35510787 PMC10084095

[2] A. Mahé , “The Practice of Skin‐Bleaching for a Cosmetic Purpose in Immigrant Communities,” Journal of Travel Medicine 21, no. 4 (2014): 282–287, 10.1111/jtm.12106. 24612323

[3] U.S. Food and Drug Administration , “FDA Works to Protect Consumers From Potentially Harmful OTC Skin Lightening Products,” 2022, https://www.fda.gov/drugs/drug‐safety‐and‐availability/fda‐works‐protect‐consumers‐potentially‐harmful‐otc‐skin‐lightening‐products.

[4] R. S. Kimyon , J. P. Schlarbaum , Y. L. Liou , et al., “Prescription‐Strength Topical Corticosteroids Available Over the Counter: Cross‐Sectional Study of 80 Stores in 13 United States Cities,” Journal of the American Academy of Dermatology 82, no. 2 (2020): 524–525, 10.1016/j.jaad.2019.10.035. 31672686

[5] K. T. Burke , M. A. Fricke , and C. M. C. DeKlotz , “Prescription‐Strength Topical Steroids Sold Without Prescription,” JAMA Dermatology 153, no. 12 (2017): 1337–1338, 10.1001/JAMADERMATOL.2017.4121. 29094143

[6] Minnesota Department of Health , “Skin Lightening Products Found to Contain Mercury, Hydroquinone and/or Steroids,” 2021, https://www.health.state.mn.us/communities/environment/skin/docs/testedprds.pdf.

